# Triglycerides to apolipoprotein A1 ratio: an effective insulin resistance-associated index in identifying metabolic dysfunction-associated fatty liver disease in type 2 diabetes mellitus

**DOI:** 10.3389/fendo.2024.1384059

**Published:** 2024-12-23

**Authors:** Wei Wang, Yang Chen, Mei Tu, Hang Ju Chen

**Affiliations:** National Metabolic Management Center, Longyan First Affiliated Hospital of Fujian Medical University, Longyan, Fujian, China

**Keywords:** triglycerides to apolipoprotein A1 ratio, triglycerides to high-density lipoprotein cholesterol ratio, triglyceride glucose index, insulin resistance, metabolic dysfunction-associated fatty liver disease, type 2 diabetes mellitus

## Abstract

**Background:**

The triglycerides to Apolipoprotein A1 ratio (TG/APOA1) holds promise to be a more valuable index of insulin resistance for the diagnosis of metabolic dysfunction-associated fatty liver disease (MAFLD) in type 2 diabetes mellitus (T2DM). This study aims to evaluate the correlation between TG/APOA1 and MAFLD, as well as compare the efficacy of TG/APOA1 with triglycerides to high-density lipoprotein cholesterol ratio (TG/HDL-c) and triglyceride-glucose (TyG) index in identifying MAFLD among individuals with T2DM.

**Method:**

This study consecutively recruited 779 individuals with T2DM for the investigation. The unenhanced abdominal CT scans were conducted to measure CT liver-spleen attenuation measurement (CT_L-S_). The CT_L-S_ less than 1.0 and without other liver comorbidities were considered to be MAFLD. The binomial logistic regression analysis and restricted cubic spines (RCS) were employed to evaluate the association between TG/APOA1 and MAFLD. The receiver operating characteristic (ROC) curve analysis was performed to compare the efficacy of TG/APOA1 with TG/HDL-c and TyG index identifying MAFLD.

**Results:**

The TG/APOA1 exhibited a substantial increase in the MAFLD group (*P<*0.05). Even after adjustments for potential confounding factors, TG/APOA1 exhibited significant associations with nonalcoholic fatty liver disease fibrosis score (*β*=0.266, *P*<0.001), fibrosis-4 index (*β*=0.123, *P*=0.029), aspartate aminotransferase-to-platelet ratio index (*β*=0.113, *P*=0.037), and CT_L-S_ (*β*=-0.225, *P*<0.001). Meanwhile, TG/APOA1 contributed to an independent variable for MAFLD, the odds ratio with a 95% CI was 2.092 (1.840-2.380) in the total population, 2.123 (1.810-2.511) in men, and 2.162 (1.824-2.587) in women. Additionally, the results also revealed a nonlinear association between elevated TG/APOA1 and higher MAFLD risk according to the RCS analysis whether in the total population, men, or women (*P* for nonlinearity and overall <0.001). Furthermore, TG/APOA1 had higher AUC level compared to TG/HDL-c and TyG index in the total population (0.769 vs 0.742, *P*=0.025; 0.769 vs 0.694, *P* < 0.001), men (0.776 vs 0.744, *P*=0.044; 0.776 vs 0.709, *P* < 0.001), and women (0.762 vs 0.728, *P*=0.041; 0.762 vs 0.674, *P* < 0.001).

**Conclusion:**

TG/APOA1 serves as an effective index of insulin resistance in identifying MAFLD, offering advantages in the screening of MAFLD in T2DM.

## Introduction

Metabolic dysfunction-associated fatty liver disease (MAFLD) represents a novel classification of nonalcoholic fatty liver disease (NAFLD), emphasizing the role of metabolic risk factors in the development and progression of NAFLD-related pathology. MAFLD is characterized by hepatic triglycerides (1) accumulation and can advance to more severe manifestations like non-alcoholic steatohepatitis (NASH), liver fibrosis, cirrhosis, and even hepatocellular carcinoma, posing a significant public health concern in the obese population (2). Notably, MAFLD demonstrates a close association with type 2 diabetes mellitus (T2DM) and obesity (3), as they share common pathophysiological mechanisms involving insulin resistance, heightened oxidative stress, perturbed hepatic glucose regulation, and impaired lipid metabolism (4–6). Furthermore, accumulating evidence has demonstrated that the impact of MAFLD extends beyond hepatic implications and profoundly influences T2DM (7). MAFLD amplifies the risk of diabetes-related complications in individuals with T2DM, including cardiovascular diseases (8) and progressive chronic kidney disease (9). Given the prevalence and detrimental effects of MAFLD in T2DM, timely identification of MAFLD and effective intervention strategies are imperative to either achieve remission or delay the progression to advanced stages.

Insulin resistance constitutes a pivotal factor in the pathogenesis of MAFLD and exacerbates its progression to nonalcoholic steatohepatitis and fibrosis (10, 11). Recent advances in MAFLD have unveiled several insulin resistance indexes that exhibit diagnostic and prognostic potential by combining lipid profiles and blood glucose parameters. Among these indexes, the TG to high-density lipoprotein cholesterol ratio (TG/HDL-c) and triglyceride-glucose (TyG) index have gained wide recognition as surrogate indicators of insulin resistance (12, 13), offering diagnostic value for MAFLD (14, 15). Apolipoprotein A1 (APOA1), the primary protein constituent of high-density lipoprotein cholesterol (HDL-c), appears to establish a potential link between MAFLD and cardiovascular disease (16). Mounting evidence suggests that APOA1 plays multifaceted roles in anti-inflammation, anti-insulin resistance, anti-atherosclerosis, and the inhibition of oxidative stress and nitric oxide production, surpassing the effects of HDL-c itself (17, 18). Additionally, an observational study revealed that the monocyte-to-APOA1 ratio outperforms the monocyte-to-HDL-c ratio in identifying metabolic syndrome (19). Given the biological properties of APOA1 and the supporting clinical evidence, it is plausible to suggest that APOA1 could serve as a more valuable lipid marker for diagnosing MAFLD. As of today, the knowledge regarding the ability of the TG to APOA1 ratio (TG/APOA1) in identifying MAFLD remains uncertain. Consequently, this study aims to evaluate the correlation between TG/APOA1 and MAFLD, as well as compare the efficacy of TG/APOA1 with other insulin resistance indexes, such as TG/HDL-c and TyG index, in identifying MAFLD among individuals with T2DM.

## Methods

### Participants

**Figure 1:**
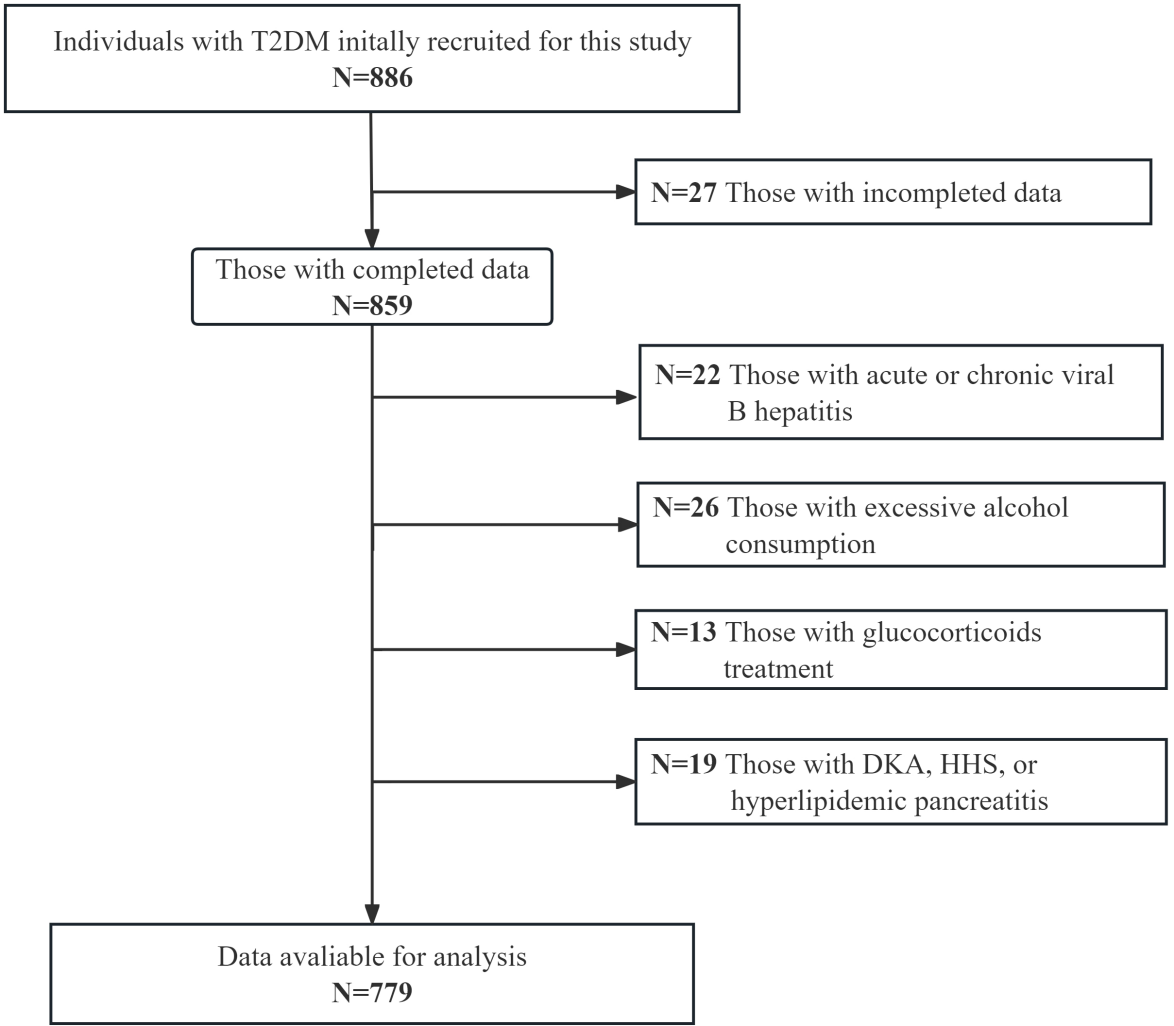
The flowchart visually depicts the process of enrolling the study population.

This cross-sectional study consecutively recruited individuals with T2DM who were admitted to the national metabolic management center at Longyan First Affiliated Hospital of Fujian Medical University from June 2022 to September 2023. Exclusion criteria were applied to eliminate individuals with the following specific conditions: (1) a history of excessive alcohol consumption (daily alcohol intake ≥ 30 g for men and ≥ 20 g for women), (2) a history of other liver comorbidities such as liver malignancy, viral hepatitis, or autoimmune hepatitis, and (3) treatment with medications that can interfere with lipid metabolism or induce liver steatosis and insulin resistance (e.g., estrogens, tamoxifen, and glucocorticoids), (4) presence of severe hyperglycemia or hypertriglyceridemia, including conditions such as diabetic ketoacidosis, hyperglycemic hyperosmolar syndrome, and hyperlipidemic pancreatitis. Before enrollment, all participants provided written informed consent, and ethical approval was obtained from the Ethical Committee of Longyan First Affiliated Hospital of Fujian Medical University (IC-2022-009). All procedures adhered to the principles outlined in the Declaration of Helsinki. Based on the requirement of a multiple binomial logistic regression model with 10-15 variables (20), following the principle of 5-10 events per variable, and considering the prevalence of MAFLD ranging from 40% to 50.0%, a sample size of 600-800 patients was planned for this study. **Figure 1** visually represents the flow chart illustrating participant recruitment in this study. Ultimately, a total of 779 participants were included in the final analysis.

### Data collection and laboratory assessments

Demographic information, encompassing sex, age, diabetic duration, smoking status, alcohol intake, medication usage, and medical history, was collected by trained research personnel using a standardized questionnaire. Additionally, anthropometric measurements, including weight, height, waist circumference (WC), and blood pressure (BP), were recorded by trained nurses upon admission. Following an overnight fast, venous blood samples were carefully obtained by the trained nurses and subsequently analyzed in the key laboratory of Longyan First Hospital. The laboratory assessments encompassed a comprehensive set of measurements comprising creatinine, alanine aminotransferase, albumin, aspartate aminotransferase (AST), uric acid (UA), FBG, serum insulin, TG, total cholesterol (TC), low-density lipoprotein cholesterol (LDL-c), APOA1, HDL-c, hemoglobin A1c (HbA1c), and platelets. The auto-biochemical analyzer (Roche Diagnostics Corporation) was used to determine the biochemical indexes. The polyethylene glycol-enhanced immunoturbidimetric assay (Maker, Chengdu, China) was used to calculate serum ApoA1 levels. HbA1c was evaluated by high-performance liquid chromatography with a D10 set (Bio-RAD).

### Definition

Insulin resistance indexes, including HOMA-IR, TG/APOA1, TG/HDL-c, and TyG index, were computed using the following equations. HOMA-IR was calculated as fasting serum insulin (µU/ml) x FBG (mmol/l)/22.5 (21). TG/APOA1 and TG/HDL-c were calculated as TG (mmol/l)/APOA1 (mmol/l) and TG (mmol/l)/HDL-c (mmol/l). TyG index was calculated as ln (TG (mg/dl) x FBG (mg/dl)/2) (22). Metabolic dysfunctions, as defined by established criteria (23), encompassed the following conditions: (1) Abdominal obesity indicated by a WC ≥90 cm in men or WC ≥ 80 cm in women. (2) Increased BP characterized by BP ≥ 130/85 mmHg or specific antihypertensive medication. (3) Elevated plasma TG levels characterized by TG≥1.70 mmol/L, or the use of specific lipid-lowering medication. (4) Low levels of HDL-c characterized by HDL-c<1.0 mmol/L in men or HDL-c<1.3 mmol/L in women, or the use of specific lipid-modifying medication. (5) Presence of prediabetes or diabetes. (6) Hyperuricemia characterized by UA ≥ 420 µmol/L or the use of specific medication targeting UA management. (7) Evidence of insulin resistance characterized by HOMA-IR ≥2.5.

### Assessment of MAFLD and liver fibrosis risk

The diagnosis of MAFLD in T2DM was established according to the latest international expert consensus statement (23), which stipulated the identification of hepatic steatosis through imaging techniques, blood biomarkers, or liver histology. In this study, the detection of fatty liver was based on the utilization of CT liver-spleen attenuation measurement (CT_L-S_), a highly accurate CT index specifically designed for evaluating fatty liver. To minimize inter-operator variability, two radiologists were involved in the assessment. CT_L-S_ was computed by dividing the mean liver attenuation by the mean spleen attenuation. According to the Guidelines for the prevention and treatment of nonalcoholic fatty liver disease (2018, China). The diagnostic criterion for fatty liver was set at CT_L-S <_1.0. Further categorization of fatty liver severity was conducted using cut-off points of 0.7 and 0.5, resulting in the classification of mild, moderate, or severe fatty liver (24).

To estimate the probability of liver fibrosis, this study utilized validated indexes known as the nonalcoholic fatty liver disease fibrosis score (NFS), the aspartate aminotransferase-to-platelet ratio index (APRI), and the fibrosis-4 (FIB-4) index, which have shown efficacy in predicting advanced liver fibrosis risk in T2DM with MAFLD (25). The NFS was calculated using the formula: -1.675 + 0.037 × age (years) + 0.094 × BMI (kg/m²) + 1.13 + 0.99 × AST/ALT ratio - 0.013 × platelet count (10^9^/L) - 0.66 × albumin (g/dL). The FIB-4 index was determined as age (years) × AST (IU/L)/(platelet count (10^9^/L) × ALT (IU/L)^1/2^). Additionally, the APRI was computed as AST (IU/L)/AST (IU/L)/platelet count (10^9^/L) × 100. Classification of MAFLD participants into low, intermediate, or high-risk groups for advanced fibrosis was based on specific cut-off points: NFS (-1.455 and 0.676), APRI (0.25 and 0.5), and FIB-4 (1.30 and 2.67).

### Statistical analysis

Statistical analysis was conducted using SPSS 26.0 software (SPSS Inc., IBM). To compare baseline characteristics between the MAFLD and Non-MAFLD groups, independent samples T-tests or Kruskal-Wallis tests were performed for continuous variables, while chi-squared (χ²) tests or Fisher’s exact tests were used for categorical variables. Correlation analyses, employing either Pearson’s correlation coefficient or Spearman’s rank-order correlation coefficient, were performed to assess associations between TG/APOA1 and liver fibrosis-related indexes, as well as CT_L-S_ in the MAFLD population. Multiple regression analysis was subsequently utilized to further analyze these correlations, with adjustments made for potential confounding variables. The influence of TG/APOA1 on the presence of MAFLD was assessed using binomial logistic regression analysis and Restricted cubic splines (RCS), controlling for relevant confounders across different models. The receiver operating characteristic (ROC) curves analysis was used to compare the identifying value of TG/APOA1 with TG/HDL-c and TyG index for MAFLD. Statistical significance was defined as a two-tailed P-value of less than 0.05, indicating a significant association or difference.

## Result

### Comparison of clinical characteristics between MAFLD and non-MAFLD group

A total of 779 participants were included in this study, with 401 (52.8%) being men. The mean age of the participants was 53.5 ± 8.0 years old. The overall prevalence of MAFLD was 51.5%. **Table 1** summarizes an overview of the comparison of clinical characteristics between the MAFLD and non-MAFLD groups. In comparison to the non-MAFLD group, various parameters exhibited significant differences in the MAFLD group. Specifically, BMI, WC, SBP, DBP, TG, UA, ALT, AST, and insulin resistance indexes such as HOMA-IR, TG/APOA1, TG/HDL-c, and TyG index. In contrast, HDL-c and APOA1 levels were decreased in the MAFLD group.

**Table 1:** Comparison of clinical characteristics between the MAFLD and non-MAFLD groups.

Variable	Total (n=779)	MAFLD (n=401)	Non-MAFLD (n=378)	*P*
Age(year)	53.5 ± 8.0	53.0 ± 7.5	54.0 ± 8.6	0.063
Men, n(%)	411 (52.8)	220 (55.1)	189 (49.7)	0.173
Smoking, n(%)	249 (32.0)	130 (34.4)	119 (29.7)	0.158
Drinking, n(%)	291 (37.4)	185(46.1)	106 (28.0)	<0.001
BMI(kg/m^2^)	24.4 ± 3.2	25.4 ± 3.2	23.3 ± 2.6	<0.001
WC (cm)	85.8 ± 7.0	88.3 ± 7.4	83.2 ± 5.8	<0.001
SBP (mmHg)	133.4 ± 18.1	139.2 ± 18.4	127.3 ± 15.6	<0.001
DBP (mmHg)	81.5 ± 9.3	83.4 ± 8.5	77.9 ± 8.7	<0.001
HbA1c(%)	8.8 ± 1.1	8.9 ± 1.1	8.8 ± 1.0	0.330
TG(mmol/L)	2.22 ± 1.36	2.64 ± 1.47	1.79 ± 1.13	<0.001
TC(mmol/L)	5.29 ± 1.22	5.20 ± 1.26	5.24 ± 1.17	0.487
HDL-c(mmol/L)	1.09 ± 0.24	0.99 ± 0.21	1.18 ± 0.24	<0.001
LDL-c(mmol/L)	3.52 ± 0.94	3.55 ± 0.98	3.49 ± 0.92	0.299
APOA1(g/L)	0.99 ± 0.19	0.70 ± 0.20	0.97 ± 0.18	<0.001
UA(umol/L)	346.3 ± 86.8	364.7 ± 91.3	326.7 ± 77.2	<0.001
Creatinine(umol/L)	69.2 ± 13.3	68.4 ± 13.3	70.1 ± 13.4	0.086
ALT (IU/L)	37.7 ± 9.0	38.6 ± 9.3	36.7 ± 8.5	0.004
AST (IU/L)	31.2 ± 7.0	32.2 ± 7.7	30.2 ± 6.1	<0.001
Albumin(g/L)	40.1 ± 4.3	39.9 ± 5.1	40.3 ± 4.1	0.783
Platelets(10^9^/L)	188.8 ± 51.6	187.8 ± 54.3	190.1 ± 49.3	0.689
HOMA-IR	3.18 ± 1.76	3.71 ± 1.81	2.62 ± 1.51	<0.001
TG/APOA1	3.00 ± 2.39	4.04 ± 2.67	1.89 ± 1.37	<0.001
TG/HDL-c	2.34 ± 1.91	2.90 ± 2.0	1.73 ± 1.59	<0.001
TyG index	9.44 ± 0.68	9.66 ± 0.64	9.21 ± 0.65	<0.001

BMI, body mass index; WC, waist circumference; HbA1c, Glycated hemoglobin; UA, uric acid; TG, triglycerides; TC, total cholesterol; HDL-c, high-density lipoprotein cholesterol; LDL-c, low-density lipoprotein cholesterol; SBP, systolic blood pressure; DBP, diastolic blood pressure; HOMR-IR, homeostasis model assessment insulin resistance; ALT, alanine aminotransferase; AST, aspartate aminotransferase; APOA1, apolipoprotein A1; TG/APOA1, triglycerides to apolipoprotein A1 ratio; TG/HDL-c, triglycerides to high-density lipoprotein cholesterol ratio; TyG, triglyceride glucose index.

### Metabolic dysfunctions and liver fibrosis risk across TG/APOA1 quartiles

**Figure 2:**
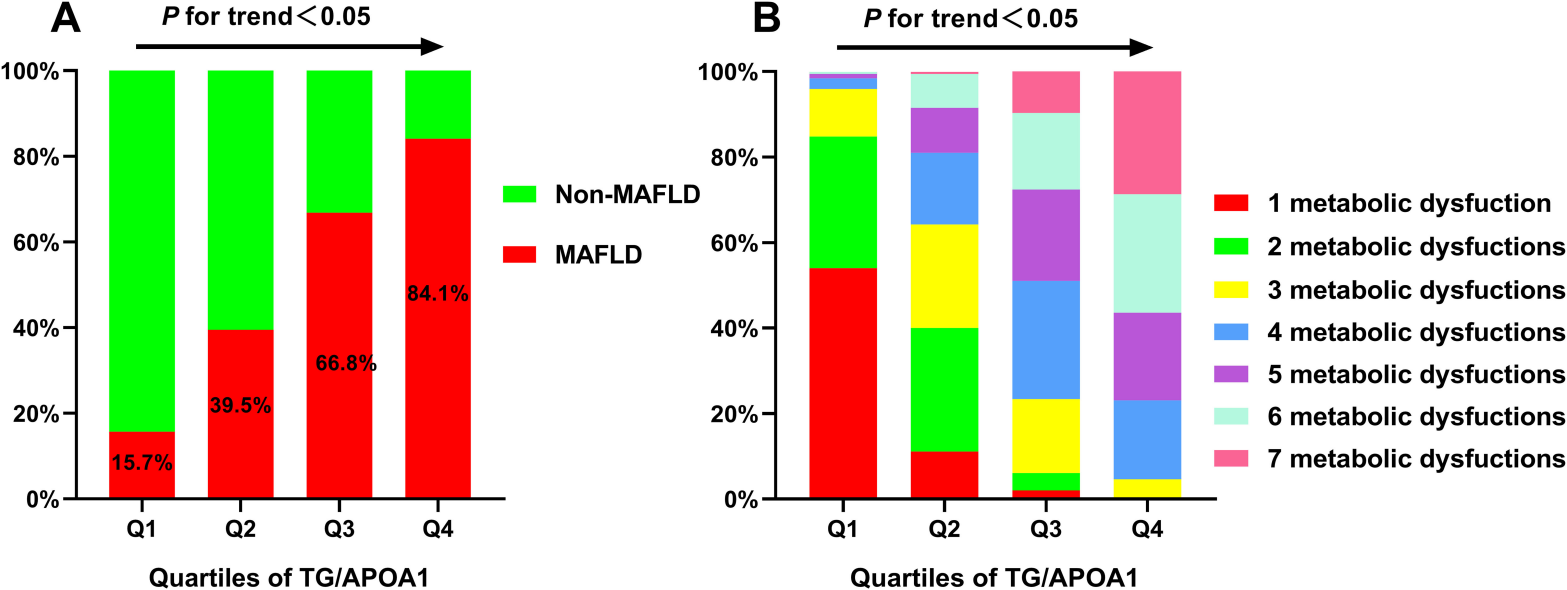
The increased prevalence of MAFLD across TG/APOA1 quartiles **(A)**. The distribution of 1 metabolic dysfunction to 7 metabolic dysfunctions in the TG/APOA quartile groups **(B)**. MAFLD, metabolic dysfunction-associated fatty liver disease; TG/APOA1, triglycerides to Apolipoprotein A1 ratio.

**Figure 3:**
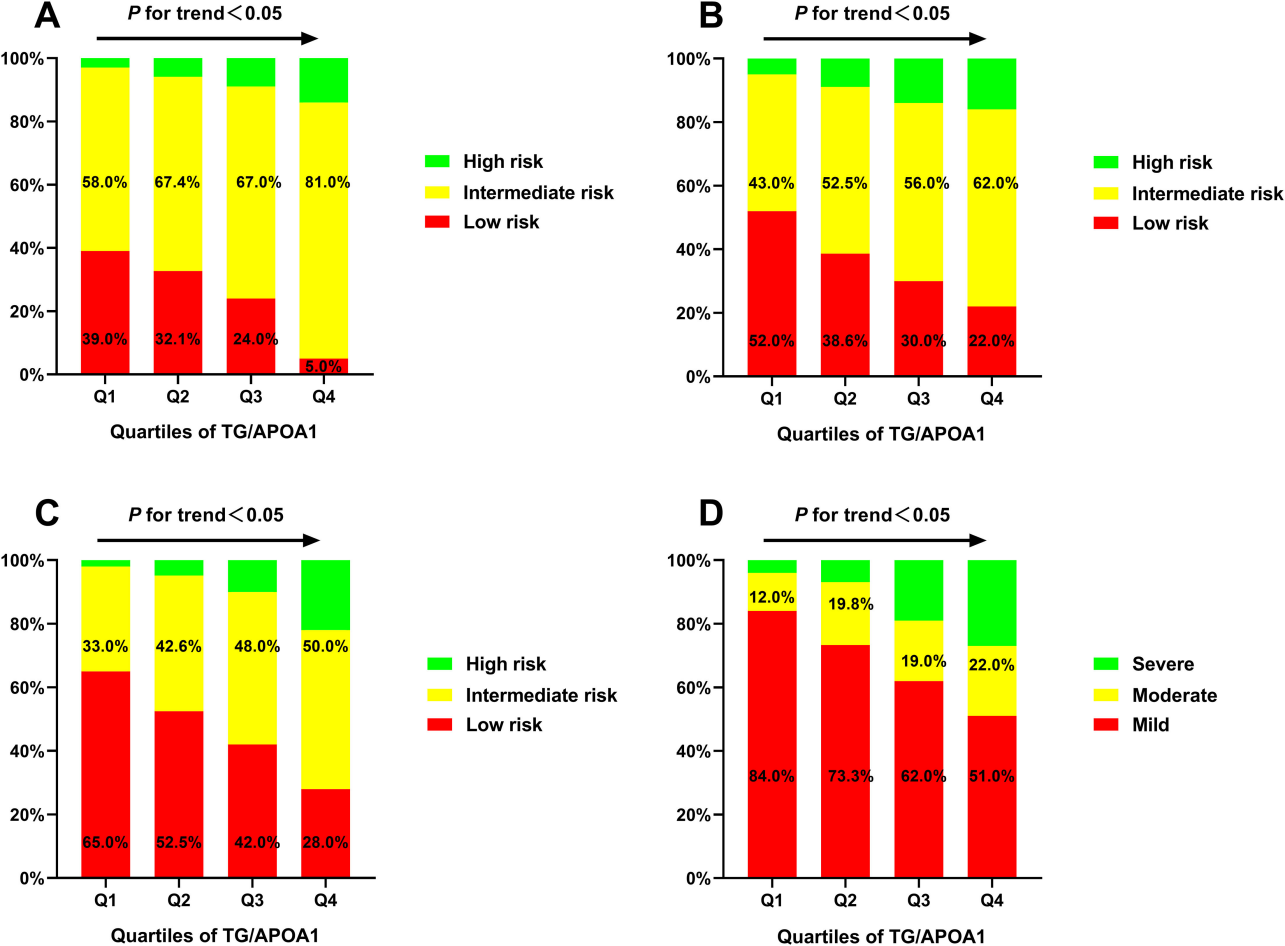
The advanced liver fibrosis risk based on the nonalcoholic fatty liver disease fibrosis score **(A)**, the fibrosis-4 index **(B)**, and the aspartate aminotransferase-to-platelet ratio index **(C)** index and the severity of fatty liver based on CT liver-spleen attenuation measurement **(D)**.

The prevalence of MAFLD and metabolic dysfunctions across TG/APOA1quartiles (Q1: <1.37; Q2: 1.37-2.31; Q3: 2.32-3.83; Q4: >3.83) in individuals with T2DM are depicted in **Figure 2**. The prevalence of MAFLD ranged from 15.7% in Q1 to 84.1% in Q4, indicating a significant increase with higher TG/APOA1 quartiles (**Figure 2A**). Furthermore, the distribution of metabolic dysfunctions differed significantly across the four quartiles (**Figure 2B**). The proportion of participants with more than three metabolic dysfunctions decreased significantly from 95.4% in the higher quartiles to 4.0% in the lower quartiles (*P* < 0.05). **Figure 3** illustrates advanced liver fibrosis risk and fatty liver severity across TG/APOA1 quartiles (Q1:<2.24; Q2:2.24-3.33; Q3:3.34-4.95; Q4:>4.95) in participants with MAFLD. The results demonstrate a significant increase in the proportion of participants with intermediate or high-risk advanced liver fibrosis in the higher quartiles compared to the lower quartiles based on NFS (**Figure 3A**), FIB-4 (**Figure 3B**), and APRI (**Figure 3C**). Additionally, higher TG/APOA1 quartiles were associated with a higher prevalence of moderate or severe fatty liver (**Figure 3D**).

### Correlations of TG/APOA1 with CT_L-S_ and advanced liver fibrosis risk

**Figure 4:**
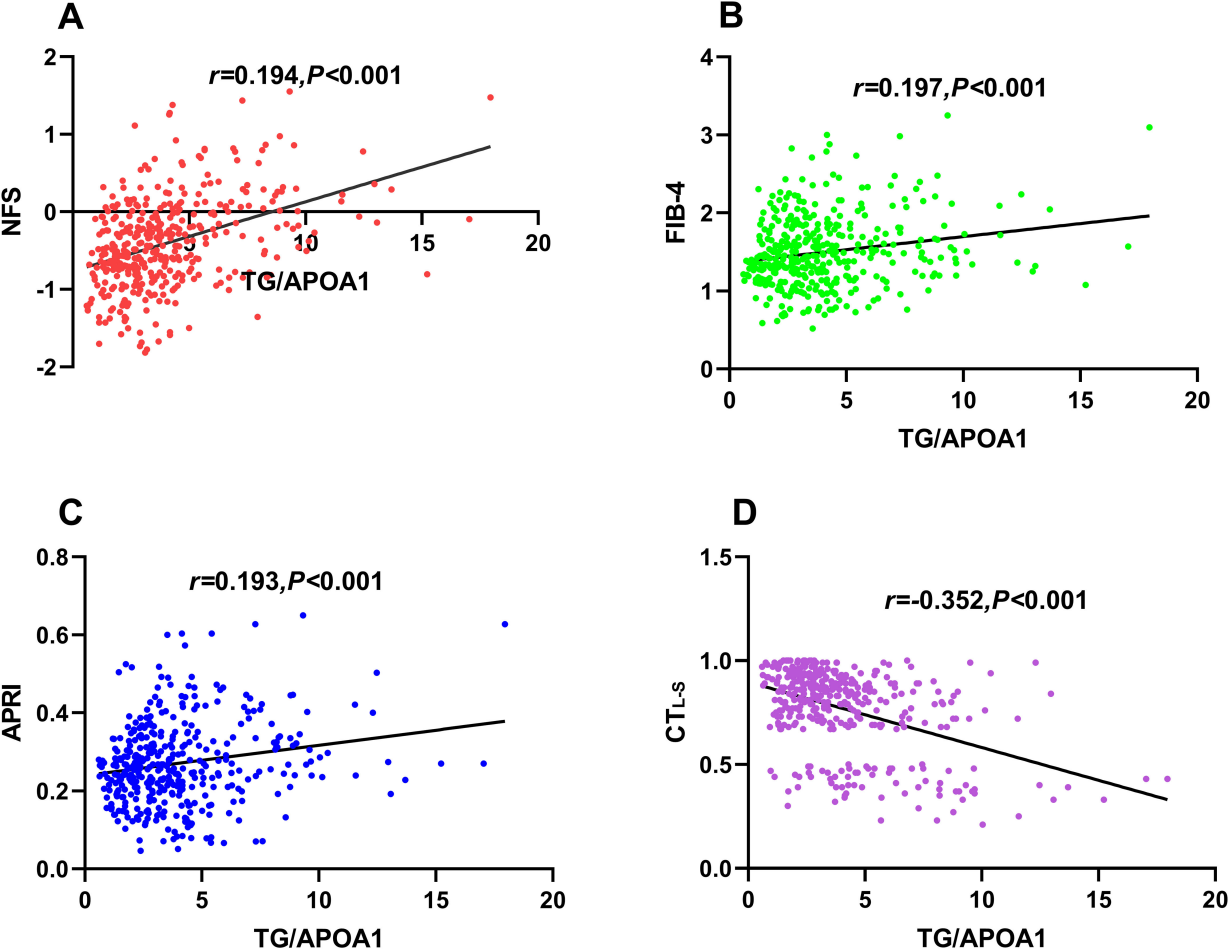
Univariate correlations between TG/APOA1 and NFS **(A)**, FIB-4 **(B)**, APRI **(C)**, and CT_L-S_ **(D)**. TG/APOA1, triglycerides to Apolipoprotein A1 ratio; NFS, nonalcoholic fatty liver disease fibrosis score; FIB-4, fibrosis-4 index **(B)**. APRI, AST-to-platelet ratio index; CT_L-S_, CT liver-spleen attenuation measurement.

**Figure 4** illustrates the univariate correlations between TG/APOA1 and NFS (**Figure 4A**), FIB-4 (**Figure 4B**), APRI (**Figure 4C**), and CT_L-S_ (**Figure 4D**) in the MAFLD population. The findings indicate positive correlations of TG/APOA1 with NFS (*r*=0.401, *P<*0.001), FIB-4 (*r*=0.197, *P<*0.001), and APRI (*r*=0.193, *P<*0.001). Conversely, a negative correlation was observed between TG/APOA1 with CT_L-S_ (*r*=-0.352, *P<*0.001). To further evaluate these correlations while accounting for potential confounding factors, multiple linear regression analyses were conducted. The results are presented in **Table 2**, demonstrating that TG/APOA1 maintained positive associations with NFS, FIB-4, and APRI while exhibiting a negative association with CT_L-S_ in Model 1 (adjustments for age, sex, diabetic duration, smoking, and drinking) and Model 2 (further adjustments for BMI, SBP, DBP, HbA1c, and UA based on Model 1). Furthermore, even after adjusting for lipid profiles such as TC, LDL, and HDL-c based on Model 2 (Model 3), TG/APOA1 continued to exhibit significant associations with NFS (*β*=0.266, *P*<0.001), FIB-4 (*β*=0.123, *P*=0.029), APRI (*β*=0.113, *P*=0.037), and CT_L-S_ (*β*=-0.225, *P*<0.001).

**Table 2:** Multivariate linear regression analysis of the association between TG/APOA1 and NFS, FIB-4, APRI, and CT_L-S_ in participants with MAFLD.

Variable	Model 1		Model 2		Model 3	
	*β*	*P*	*β*	*P*	*β*	*P*
NFS	0.388	<0.001	0.323	<0.001	0.266	<0.001
FIB-4	0.193	<0.001	0.147	0.005	0.123	0.029
APRI	0.191	<0.001	0.132	0.012	0.113	0.037
CT_L-S_	-0.329	<0.001	-0.253	<0.001	-0.225	<0.001

Model 1: adjusted for age, gender, diabetic duration, drinking, and smoking.

Model 2: further adjustment for body mass index, waist circumference, systolic blood pressure, diastolic blood pressure, glycated hemoglobin A1c, and uric acid.

Model 3: additional adjustment for lipid profiles like total cholesterol, low-density lipoprotein cholesterol, and high-density lipoprotein cholesterol.

TG/APOA1, triglycerides to Apolipoprotein A1 ratio; NFS, nonalcoholic fatty liver disease fibrosis score; FIB-4, fibrosis-4 index; APRI, AST-to-platelet ratio index; MAFLD, metabolic dysfunction-associated fatty liver disease.

### Correlations of TG/APOA1 with MAFLD

**Figure 5:**
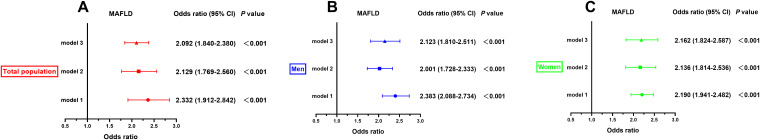
The correlation of TG/APOA1 with MAFLD after adjusting for different models in the total population **(A)**, men **(B)**, and women **(C)**. model 1: adjustments for age, sex, diabetic duration, smoking, and drinking. model 2: further adjustments for metabolic dysfunctional indicators like body mass index, waist circumference, systolic blood pressure, diastolic blood pressure, glycated hemoglobin A1c, uric acid, total cholesterol, low-density lipoprotein cholesterol, and high-density lipoprotein cholesterol based on model 1. model 3: further adjusting for liver functional indicators like aminotransferase, aspartate aminotransferase albumin, albumin, and platelets based on model 2. MAFLD, metabolic dysfunction-associated fatty liver disease; TG/APOA1, triglycerides to Apolipoprotein A1 ratio.

**Figure 6:**
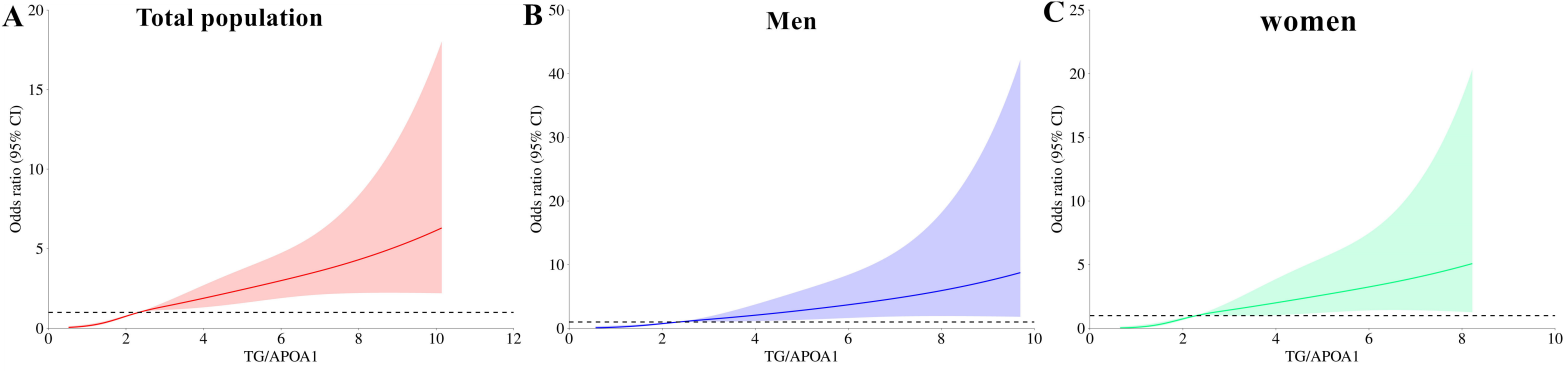
Restricted cubic spines analysis of the correlation between TG/APOA1 and MAFLD after adjusting for model 3 in the total population **(A)**, men **(B)**, and women **(C)**. model 3: adjustments for age, sex, diabetic duration, smoking, drinking, body mass index, waist circumference, systolic blood pressure, diastolic blood pressure, glycated hemoglobin A1c, uric acid, total cholesterol, low-density lipoprotein cholesterol, high-density lipoprotein cholesterol, and liver functional indicators like aminotransferase, aspartate aminotransferase albumin, albumin, and platelets MAFLD, metabolic dysfunction-associated fatty liver disease; TG/APOA1, triglycerides to Apolipoprotein A1 ratio.

**Figure 5** illustrates the correlation between TG/APOA1 and MAFLD following adjustments for confounding factors using binomial logistic regression analysis. The findings reveal that TG/APOA1 demonstrated an independent correlation with MAFLD in model 1(adjustments for age, sex, diabetic duration, smoking, and drinking) and model 2(further adjustments for metabolic dysfunctional indicators like BMI, WC, SBP, DBP, HbA1c, UA, TC, LDL, and HDL-c based on model 1) whether in the total population, men, or women. Notably, even after further adjusting for liver functional indicators like ALT, AST, albumin, and platelets based on model 2 (model 3), TG/APOA1 remained significantly associated with MAFLD. The OR with a 95% CI was calculated as 2.092 (1.840-2.380) in the total population, 2.123 (1.810-2.511) in men, and 2.162 (1.824-2.587) in women. **Figure 6** illustrates the relationship between TG/APOA1 and MAFLD analyzed using RCS. The results indicate a nonlinear increasing association between TG/APOA1 and MAFLD even after adjustments for model 3 whether in the total population (**Figure 6A**), men (**Figure 6B**), or women (**Figure 6C**). Crucially, both the statistical values for nonlinearity and overall association are below the threshold of significance (*P* < 0.001).

### Comparison of MAFLD identifying value between TG/APOA1 and TG/HDL-c, TyG index

**Figure 7:**
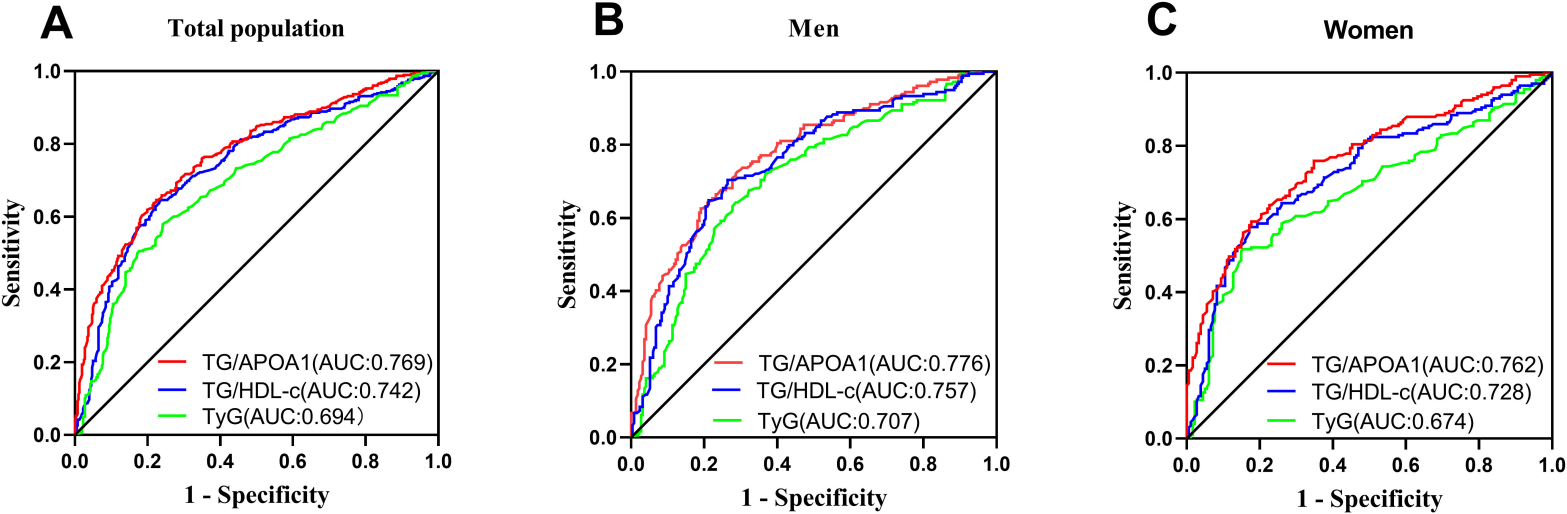
Comparison of MAFLD identifying value between TG/APOA1 and TG/HDL-c, TyG index in the total population **(A)**, men **(B)**, and women **(C)**. MAFLD, metabolic dysfunction-associated fatty liver disease; TG/APOA1, triglycerides to Apolipoprotein A1 ratio; TG/HDL-c, triglycerides to high-density lipoprotein cholesterol ratio; TyG index, triglyceride-glucose index.

**Figure 7** illustrates the comparative efficacy of TG/APOA1, TG/HDL-c, and the TyG index in identifying MAFLD. In the total population (**Figure 7A**), TG/APOA1 demonstrated a superior AUC compared to TG/HDL-c (0.769 vs 0.742, *P*=0.025) and the TyG index (0.769 vs 0.694, *P* < 0.001). Among men (**Figure 7B**), TG/APOA1 also exhibited a higher AUC than TG/HDL-c (0.776 vs 0.744, *P*=0.044), and the TyG index (0.776 vs 0.709, *P* < 0.001). Similarly, in women (**Figure 7C**), TG/APOA1 displayed a higher AUC level than TG/HDL-c (0.762 vs 0.728, *P*=0.041), and the TyG index (0.762 vs 0.674, *P* < 0.001).

## Discussion

MAFLD has emerged as a prevalent cardiometabolic disorder with far-reaching effects beyond the liver, including its impact on T2DM. Consequently, early identification of high-risk MAFLD cases in T2DM becomes imperative for timely intervention. Notably, recent evidence suggests that APOA1 plays a pivotal role in linking MAFLD to cardiovascular disease and possesses multifaceted functions in anti-inflammation and anti-insulin resistance, surpassing the influence of HDL-c alone. Therefore, the TG/APOA1 holds promise to be a more valuable insulin resistance marker for diagnosing MAFLD. This study evaluated the correlation between TG/APOA1 and MAFLD and compared the efficacy of TG/APOA1 with TG/HDL-c and TyG index in identifying MAFLD among individuals with T2DM. The results revealed that TG/APOA1 exhibited significant associations with NFS, FIB-4, APRI, and CT_L-S_. Meanwhile, TG/APOA1 contributed to an independent variable for MAFLD. This study also uncovered a notable association between elevated TG/APOA1 and higher MAFLD risk. Notably, TG/APOA1 outperformed both TG/HDL-c and the TyG index in identifying MAFLD among individuals with T2DM.

The intricate relationship between metabolic dysfunctions and the complex mechanisms underlying the development of NAFLD has prompted the consideration of renaming it as MAFLD (26). Aside from liver damage, MAFLD is frequently accompanied by various metabolic dysfunctions, including obesity, hypertension, dyslipidemia, hyperuricemia, and increased insulin resistance (27). Hence, this study observed that participants in the MAFLD group exhibited higher levels of WC, SBP, DBP, TG, UA, and HOMA-IR. Conversely, participants in the MAFLD group exhibited lower levels of HDL-c and APOA1 compared to the non-MAFLD group. Insulin resistance, a central contributor to MAFLD, disrupts glucose and lipid metabolism, thereby driving the onset and progression of the disease (10). Excessive hepatic triglyceride accumulation characterizes MAFLD, and hepatic insulin resistance contributes to impaired inhibition of hepatic gluconeogenesis and increased *de novo* lipogenesis, triggering hepatic lipogenesis and impaired glucose metabolism (1, 28), the combination of TG with HDL-c or FBG as insulin resistance indexes of MAFLD in T2DM has been explored in previous studies. Zhu. et al. identified a robust association between TG/HDL-c and the risk of NAFLD in a cohort of 1913 participants with T2DM (29). Additionally, Malek M. et al. delineated independent correlations between the TyG index and NAFLD in a study involving 175 individuals with T2DM (30). Consistent with the aforementioned studies, this study revealed that TG/APOA1 contributed to an independent variable for MAFLD even after adjusting for metabolic and liver functional profiles. This study also uncovered a notable association between elevated TG/APOA1 and higher MAFLD risk from the RCS analysis. The findings indicated that TG/APOA1 contributed to being an independent risk factor for MAFLD and holds promise to be a valuable insulin resistance marker for identifying MAFLD.

Liver biopsy was conventionally regarded as the gold standard for diagnosing NAFLD and liver fibrosis, representing a primary endpoint in clinical studies. However, its invasive nature and significant variability have restricted its widespread clinical application (31). Consequently, several non-invasive methods, including advanced imaging techniques and blood biomarkers, have emerged in recent decades as alternatives to liver biopsy. Notably, NFS, FIB-4, and APRI have been validated and recommended as indexes to assess the risk of advanced liver fibrosis in MAFLD (25). Previous studies have reported a positive correlation between the TG/HDL-c and NFS, as well as FIB-4, among 265 participants with NAFLD (32). Similarly, Ting et al. identified TG/HDL-c as an independent predictor of liver fibrosis evaluated through controlled attenuation parameters and liver stiffness measurements in pediatric NAFLD (33). This study aligns with these observations regarding TG/HDL-c, demonstrating a positive and independent association between TG/APOA1 and NFS, FIB-4, and APRI. Consequently, it can be speculated that TG/HDL-c may play a role in the development and progression of MAFLD. However, further follow-up studies incorporating liver biopsies are warranted to confirm this hypothesis. Additional investigations are necessary to gather more evidence through longitudinal examination, ultimately strengthening the relationship between TG/APOA1 and liver biopsies. This comprehensive approach will facilitate validating the potential of TG/APOA1 as a reliable marker in assessing the risk of advanced liver fibrosis in MAFLD.

Emerging evidence has suggested that the TG/HDL-c and TyG index hold diagnostic value for MAFLD in individuals with T2DM (34, 35). However, compared to TG/APOA1 and TG/HDL-c, the TyG index has certain limitations in diagnosing cardiometabolic diseases. Some studies have not definitively confirmed the close association between the TyG index and cardiovascular events, particularly in diabetic patients who may experience extreme FBG levels (36). This study compared the efficacy of TG/APOA1 with TG/HDL-c and TyG index in identifying MAFLD among individuals with T2DM. The results demonstrated that TG/APOA1 and TG/HDL-c outperformed the TyG index in identifying MAFLD. Additionally, TG/APOA1 exhibited superior identification capability for MAFLD compared to TG/HDL-c. Notably, the superiority of TG/APOA1 over TG/HDL-c in identifying MAFLD may be attributed to the biological properties of APOA1. Beyond its well-documented cardioprotective function, recent research has shown that APOA1 also plays novel roles in mitigating inflammation and insulin resistance in the pathogenesis of NAFLD. Among the transcription factors involved in fatty acid metabolism, inflammation, and fibrosis in MAFLD, peroxisome proliferator-activated receptors (PPARs) play crucial roles, exhibiting high oxidative rates in the liver (37). Chen et al. identified APOA1 as a central protein linking PPARs and NAFLD, as it beneficially regulates 16 out of 21 upstream regulators involved in NAFLD (38). Additionally, the anti-inflammatory function of HDL-c has been demonstrated to depend on its functionality rather than serum level, particularly in individuals with T2DM (39). Considering that APOA1 is the functional protein component of HDL-c, serum levels of APOA1 may better reflect the anti-insulin properties of HDL-c.

### Strength and limitation

This study possesses noteworthy strengths, primarily contributing to a novel insulin resistance index for diagnosing MAFLD in T2DM. In addition, this study also conducted the RCS analysis and adjusted the potential confounders to evaluate the association between TG/APOA1 and MAFLD. However, it is important to acknowledge several limitations associated with this investigation. Firstly, hepatic steatosis assessment was conducted through unenhanced CT scans, rather than utilizing liver biopsy, which is considered the gold standard criteria. This methodological variation may introduce potential discrepancies in the accuracy of the diagnosis. Secondly, the study design was cross-sectional, lacking a longitudinal follow-up, limiting the ability to establish a direct correlation between TG/APOA1 and MAFLD over time. Future studies with a prospective approach allowing for longitudinal evaluations are warranted to explore the dynamic relationship between TG/APOA1 and the progression of MAFLD. Lastly, it is worth noting that the data for this study were collected exclusively from a single center within the Chinese population. Consequently, caution should be exercised when generalizing the findings to other populations due to potential variations related to race and ethnicity.

## Conclusion

In conclusion, this study demonstrated significant associations between TG/APOA1 and NFS, FIB-4, APRI, and MAFLD. Importantly, TG/APOA1 exhibited superior diagnostic capability for identifying MAFLD compared to TG/HDL-c and the TyG index. These findings suggest that TG/APOA1 may be an effective index in identifying MAFLD. Overall, these results highlight the potential of TG/APOA1 as a promising index for efficient screening of MAFLD.

## Data Availability

The original contributions presented in the study are included in the article/supplementary material. Further inquiries can be directed to the corresponding author.
